# Influence of Content of Al_2_O_3_ on Structure and Properties of Nanocomposite Nb-B-Al-O films

**DOI:** 10.1186/s11671-015-1149-z

**Published:** 2015-11-24

**Authors:** Na Liu, Lei Dong, Lei Dong, Jiangang Yu, Yupeng Pan, Rongxin Wan, Hanqing Gu, Dejun Li

**Affiliations:** Energy & Materials Engineering Centre, College of Physics and Materials Science, Tianjin Normal University, Tianjin, 300387 China; Tianjin Institute of Urological Surgery, Tianjin Medical University, Tianjin, 300070 China

**Keywords:** Nb-B-Al-O nanocomposite films, Crystallization, Amorphous, Mechanical, Bombarding energy

## Abstract

Nb-B-Al-O nanocomposite films with different power of Al_2_O_3_ were successfully deposited on the Si substrate via multi-target magnetron co-sputtering method. The influences of Al_2_O_3_’s content on structure and properties of obtained nanocomposite films through controlling Al_2_O_3_’s power were investigated. Increasing the power of Al_2_O_3_ can influence the bombarding energy and cause the momentum transfer of NbB_2_. This can lead to the decreasing content of Al_2_O_3_. Furthermore, the whole films showed monocrystalline NbB_2_’s (100) phase, and Al_2_O_3_ shaded from amorphous to weak cubic-crystalline when decreasing content of Al_2_O_3_. This structure and content changes were proof by X-ray diffraction (XRD) and high-resolution transmission electron microscopy (TEM) and X-ray photoelectron spectroscopy (XPS). When NbB_2_ grains were far from each other in lower power of Al_2_O_3_, the whole films showed a typical nanocomposite microstructure with crystalline NbB_2_ grains embedded in a matrix of an amorphous Al_2_O_3_ phase. Continuing increasing the power of Al_2_O_3_, the less content of Al_2_O_3_ tended to cause crystalline of cubic-Al_2_O_3_ between the close distances of different crystalline NbB_2_ grains. The appearance of cubic-crystallization Al_2_O_3_ can help to raise the nanocomposite films’ mechanical properties to some extent. The maximum hardness and elastic modulus were up to 21.60 and 332.78 GPa, which were higher than the NbB_2_ and amorphous Al_2_O_3_ monolithic films. Furthermore, this structure change made the chemistry bond of O atom change from the existence of O-Nb, O-B, and O-Al bonds to single O-Al bond and increased the specific value of Al and O. It also influenced the hardness in higher temperature, which made the hardness variation of different Al_2_O_3_ content reduced. These results revealed that it can enhance the films’ oxidation resistance properties and keep the mechanical properties at high temperature. The study highlighted the importance of controlling the Al_2_O_3_’s content to prepare well-defined films with high mechanical properties and thermal stability.

## Background

Transition metal boride thin films are used for a wide variety of applications as they combine excellent mechanical properties with high thermal stability, oxidation, and corrosion resistance properties [1–3]. Directional covalent bonding of boron atoms and high electron concentrations introduced by transition metals are considered as two essential parameters for designing better mechanical properties materials [4, 5]. Considering the similar lattice constant with transition metal boride, NbB_2_ is endowed with excellent mechanical properties such as wear resistance and chemical inertness [6–8]. Compared to other boride, NbB_2_’s melting point is more than 3000 °C, appearing to be a promising high-temperature-resistant material. Due to these properties, NbB_2_ films have been considered as protective coatings for applications.

The incorporation of transition metal boride particulates improves the properties of ceramic matrix composites in terms of mechanical strength, abrasion, and wear of the composite of cutting tools in industrial applications [9–11]. Furthermore, cutting tools working in extreme conditions such as high temperature must possess thermostability and oxidation resistance. Different from boride, alumina is more applicable for its oxidation resistance, which becomes the new demand for cutting tools. The addition of borides to alumina is expected to result in a high mechanical strength and good oxidation resistance [12–14]. In our precious work, we found that the combination of Al_2_O_3_ and NbB_2_ can produce an improvement in films’ mechanical, thermal stability, and resistance properties [15]. However, the detailed influence of content and structure was not clear enough. Considering the hardness of Al_2_O_3_ monolithic film is much less than NbB_2_, we choose to control the content of Al_2_O_3_ to further research the Nb-B-Al-O nanocomposite films’ structure and properties.

In this paper, Nb-B-Al-O nanocomposite films were synthesized by multi-target magnetron co-sputtering NbB_2_ and Al_2_O_3_ targets. The power of Al_2_O_3_ were changed in order to explore their content’s effect on the microstructure and mechanical properties of Nb-B-Al-O films and to find an optimum composition that yield both high hardness and low friction coefficient. An important aim of this study is to investigate the change of Al_2_O_3_’s content distribution in the structure of nanocomposite films with XRD and TEM and correlate the mechanical properties to these structure changes. Finally, the effect on structure, orientation, mechanical, thermal stability, and oxidation resistance properties of nanocomposite films were discussed based on different power of Al_2_O_3_.

## Methods

The nanocomposite Nb-B-Al-O films were synthesized on Si substrate by a computer controllable magnetron sputtering system. The NbB_2_ and Al_2_O_3_ (both 99.9 % purity) targets were respectively connected to DC-pulsed and RF source sputter guns, wh ich were fixed at both sides of the chamber. The base pressure of experiment was lower than 4.0 × 10^−4^ Pa. High purity argon (99.999 %) was used as the sputtering gas in order to deposit films. Si substrates were cleaned in an ultrasonic agitator in acetone and ethanol for 15 min and dried using compressed air before being placed into the chamber. Subsequently, the substrates were sputter-cleaned for 15 min at −600 V bias voltages and a pressure of 5.0 Pa in chamber prior to nanocomposite deposition. The deposition process was carried on at an Ar gas flow of 40 sccm and a work pressure of 0.5 Pa. We keep NbB_2_ target deposited at power of 120 W and different deposition powers of Al_2_O_3_ were changed from 40 to 160 W at room temperature with −100 V bias voltage. Furthermore, a little temperature test was performed to compare the thermostability in different content of Al_2_O_3_, and the detail process of substrate treatment was previously reported [15].

Scanning electron microscope (SEM, SU8010, Hitachi, Japan) was used to observe the fracture surface morphologies of the Nb-B-Al-O films. And, the surface topography was observed by a JEM-2100 electron microscope (TEM). Wide angle X-ray diffraction (XRD) was employed for the determination of the films’ structure and crystalline nature. An X-ray photoelectron spectrometer (XPS) was used to characterize the chemical composition and chemical bonds; the contaminated C1s (284.6 eV) was used as a reference for correcting charge shift. The XPS spectra were fitted by XPSPEAK software, and Shirley background was chosen for background calculation of the XPS spectra. The Ambios XP-2 surface profilometer was used to measure thickness and the residual stress of coatings, which was calculated by the Stoney equation [16]. Nano Indenter XP system measured hardness and modulus of the films. The properties were evaluated using the Oliver and Pharr’s analysis technique [17], this technique allowed to measure the contact stiffness as well as the load and displacement at any point on the loading curve. This system was also used to perform nanoscratch test.

## Results and Discussion

**Fig. 1:**
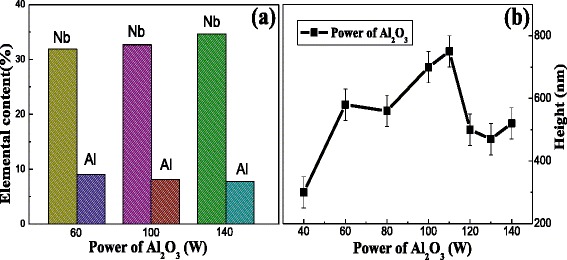
Nb and B elemental composition (at.%) EDX of Nb-B-Al-O nanocomposite films at different power of Al_2_O_3_ (**a**) and height of Nb-B-Al-O nanocomposite films at different power of Al_2_O_3_ (**b**)

Figure 1a shows the elemental composition (at.%) energy-dispersive X-ray spectroscopy (EDX) from XPS of the Nb-B-Al-O nanocomposite films prepared at 120 W sputtering power of NbB_2_ and different sputtering power of Al_2_O_3_. With the increasing sputtering power of Al_2_O_3_, its content decreases. The total content of NbB_2_ is much higher than Al_2_O_3_’s. It is considered keeping the influence of NbB_2_’s mechanical properties is less affected by Al_2_O_3_. This EDX result from XPS is in agreement with the EDX from SEM, which is not shown here. Then, we kept the deposition time that is 2 h and measured the height of different power of Al_2_O_3_ by XP-2 surface profilometer (see from Fig. 1b). While increasing power of Al_2_O_3_, the deposition rate of film is also changed as it increases first before it decreases again. Considering the change trend of thickness, the film’s rate is not just determined by content of Al_2_O_3_ but also other factors changed in the nanocomposite films.

**Fig. 2:**
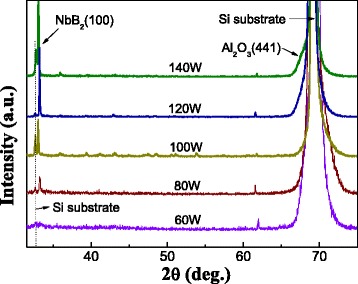
XRD patterns of Nb-B-Al-O nanocomposite at different power of Al_2_O_3_

Figure 2 shows the XRD diffractograms from the nanocomposite films with different powers of Al_2_O_3_. The amorphous character of the Al_2_O_3_ and NbB_2_ monolithic film is also indicated in XRD pattern, which is not shown here. It was observed that all the films’ diffraction peaks can be assigned to monocrystalline NbB_2_ with (100) preferred orientation. At higher content of Al_2_O_3_, the monocrystalline NbB_2_ shows only a broad feature centered at about 34°, which has a weak crystallization, and the film’s grain size is small. The high content (40 and 60 W power) of Al_2_O_3_ is similar to each other without any clear crystalline peak of nanocomposite film in Fig. 2. With the decreasing of the Al_2_O_3_’s content, the peak of NbB_2_ (100) is became shaper, the whole film showed better crystalline (seen from 80 and 100 W images). TEM results also confirmed similar. When the power of Al_2_O_3_ reaches 120 W, the intensity peak of NbB_2_ (100) is continuing to increase to its maximum. But continuing to increase the power of Al_2_O_3_ to 140 W, the (100) peak is a little decreased. Furthermore, when the Al_2_O_3_’s power is higher than 120 W, there appears (441) crystal plane of cubic Al_2_O_3_. And, 140-W power film has more clear (441) peak, which is close to the Si substrate, and the strong substrate peak is unsymmetrical. It can be explained by that when the content of Al_2_O_3_ is decreased to some constant, it appears to crystalline.

**Fig. 3:**
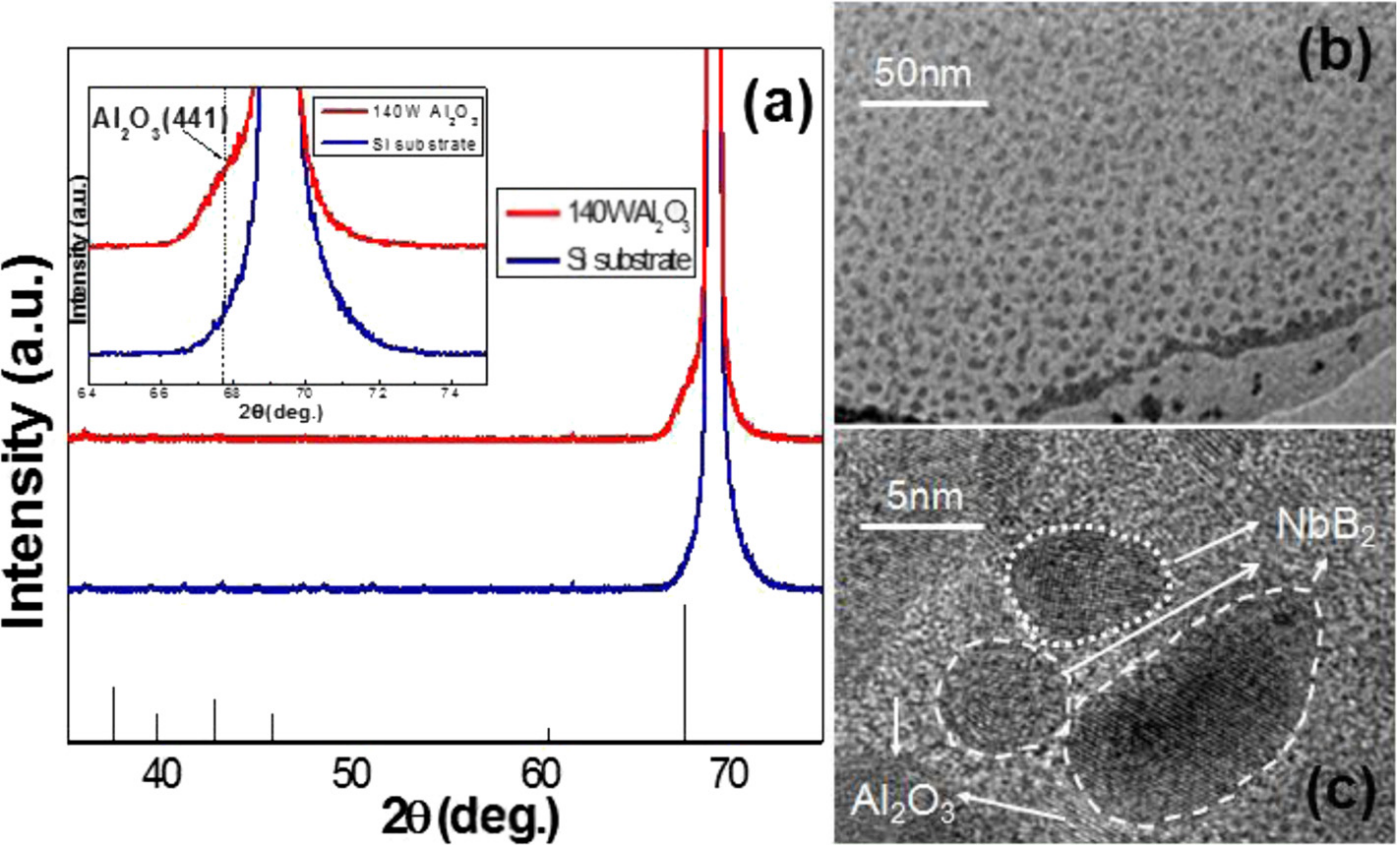
XRD patterns of Nb-B-Al-O nanocomposite film at 140 W power of Al_2_O_3_ (**a**), TEM images taken at low (**b**) and high (**c**) magnification of 140 W power of Al_2_O_3_

The base peak of Al_2_O_3_ can be assigned to cubic (NaCl structure) with a lattice parameter of 7.95 Å. When we compared with Si substrate, we can find a clear peak of c-Al_2_O_3_’s (441), which is the main peak of cubic-crystallization Al_2_O_3_. When increasing the power of Al_2_O_3_, the nanocomposite film appears weak crystalline, and this result is in agreement with TEM images in Fig. 3. The light part represents Al_2_O_3_ and the dark part represents NbB_2_. The whole nanocomposite films show a typical nanocomposite microstructure with crystalline NbB_2_ grains embedded in a matrix of an amorphous Al_2_O_3_ phase from the low magnification image. When the content of Al_2_O_3_ is decreased to some extent, we can find the light part in the interface of Al_2_O_3_ and NbB_2_ appears crystallization from high magnification image. We can conclude that the decreasing Al_2_O_3_ content in nanocomposite films changes its structure from amorphous to weak crystalline in the interfaces of particle NbB_2_ and Al_2_O_3_. The film’s rate of deposition increased in low power of Al_2_O_3_ because less Al_2_O_3_ content can promote the film’s growth. We measured the Al_2_O_3_ and NbB_2_ monolithic films’ deposition rate and found that the rate of NbB_2_ is much higher than Al_2_O_3_. Then, the appearance of Al_2_O_3_’s crystalline plane in high power makes the films densification in the interface and its rate decreased. Besides that, the bombarding energy caused by increasing power of Al_2_O_3_ can become another important factor of deposition rate changes. A proper bombarding energy of lower power is beneficial in promoting the deposition of NbB_2_ and Al_2_O_3_. However, the high bombarding energy in higher power of Al_2_O_3_ tends to cause radiation damage [18]. And, more energetic ions and high-energy electrons will bombard newly formed film. Re-sputtering process of the deposited films will lead to the decrement of film thickness [19]. In order to eliminate the effect of the film’s thickness, we keep the thickness of nanocomposite Nb-B-Al-O films in different power of Al_2_O_3_ at 500–600 nm to explore its properties.

**Fig. 4:**
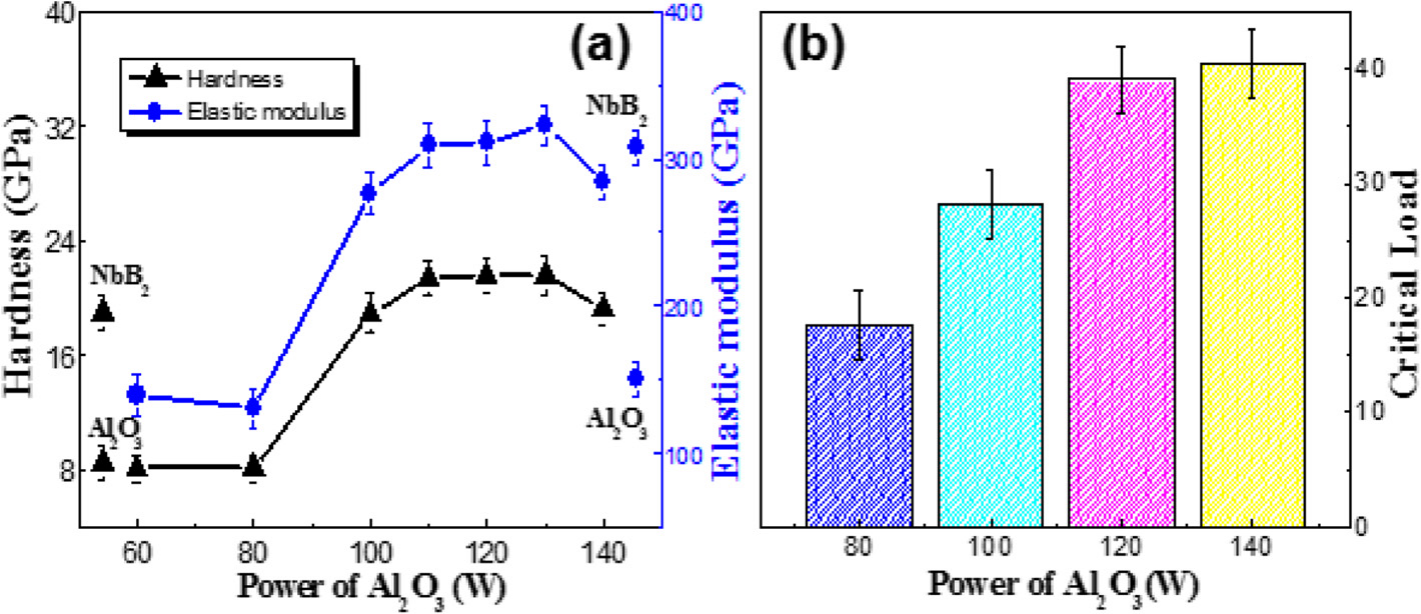
Hardness and elastic modulus (**a**), critical load (**b**) of the Nb-B-Al-O nanocomposite films at different power of Al_2_O_3_

Figure 4a shows the variations of hardness and elastic modulus as different power of Al_2_O_3_ in the 120 W power of NbB_2_, together with hardness and elastic modulus of NbB_2_ and Al_2_O_3_ monolithic films. With increasing the power of Al_2_O_3_ to 140 W, the hardness of the nanocomposite films increases first before it decreases again. The elastic modulus of the samples also followed the similar trend as that of hardness. In order to make sure the accuracy results of hardness, we also measured the 110 and 130 W power of Al_2_O_3_; the trend of hardness change is still remaining stability. The maximum hardness and elastic modulus are up to 21.60 and 332.78 GPa, which are higher than the NbB_2_ and Al_2_O_3_ monolithic films and keep constant in 110 to 130 W power of Al_2_O_3_. Figure 4b shows the critical fracture load of Nb-B-Al-O films with power of Al_2_O_3_. We can, through the critical fracture load Lc, characterize the adhesion strength of the film or the film’s fracture resistance. Further, other factors such as inherent internal stress, hardness, and plastic recovery can also influence the film’s fracture resistance. Just like the trend of hardness, the Lc of nanocomposite films is increased with the increasing power of Al_2_O_3_ and then remains constant.

**Fig. 5:**
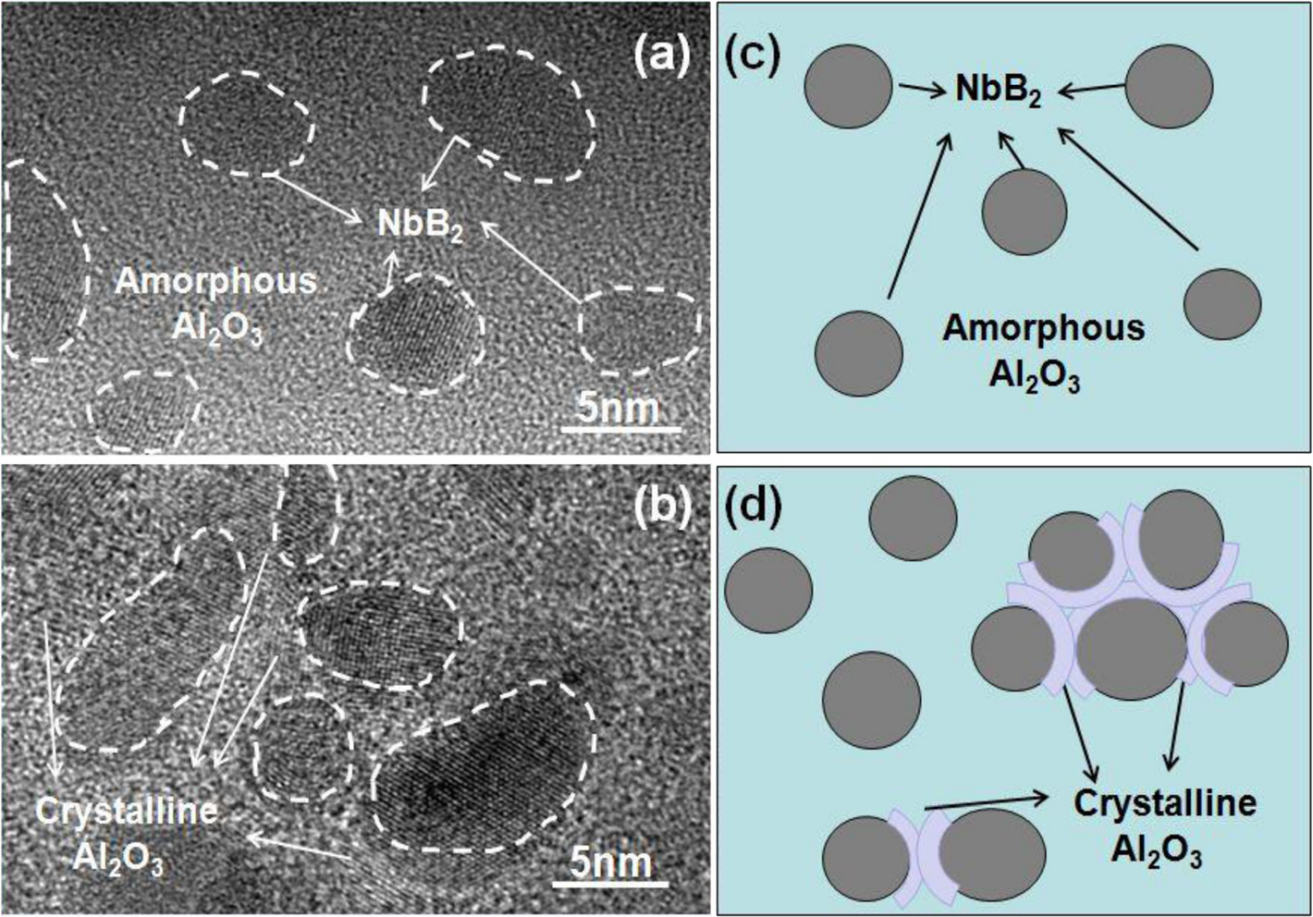
TEM images taken at high magnification of 60 W (**a**), 140 W (**b**) power of Al_2_O_3_, and its model representation (**c**, **d**)

From earlier research [20, 21], nanocomposite films also show hardness enhancement compared to monolithic films. Our results showed that the interfaces in Al_2_O_3_ and NbB_2_ play a leading role to its mechanical properties. When increasing the power of Al_2_O_3_, the content of Al_2_O_3_ is decreased because of higher bombarding energy’s radiation damage. The decreasing Al_2_O_3_ makes the distance of NbB_2_ grains close to each other, and the size of crystallization NbB_2_ grains are increased (see from Fig. 5). The films showed a typical nanocomposite microstructure with crystalline NbB_2_ grains embedded in a matrix of an amorphous Al_2_O_3_ phase in higher content of Al_2_O_3_. Continuing increasing the power of Al_2_O_3_, there appear crystalline of cubic-Al_2_O_3_ between the close distances of different crystalline NbB_2_ grains in some area. The appearance of cubic-crystallization Al_2_O_3_ can help to raise the nanocomposite films’ mechanical properties to some extent. The interfaces of NbB_2_ and Al_2_O_3_ act as barriers to the motion of dislocations glide in nanocomposite film when explain the mechanisms of promoted hardness [22]. Furthermore, the dislocation blocking due to coherency strains for different nanocrystalline grains also makes a contribution to hardness enhancement [23].

**Fig. 6:**
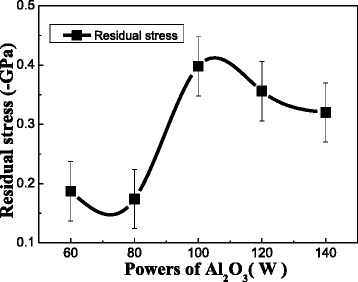
Residual stresses of Nb-B-Al-O nanocomposite films at different power of Al_2_O_3_

The compressive stress of the nanocomposite films determined by XP-2 profiler is influenced by the power of Al_2_O_3_ as is shown in Fig. 6. Residual stress is generated during the coating growth process. High residual stress (σ) is the main reason for film delamination and plastic deformation. Therefore, the reduced residual stress in films is a key factor for these films to explore more applications. All the films’ residual stress is less than 1 GPa. It is due to introduction of Al_2_O_3_ into crystalline phase NbB_2_ that helps to relax the stress. The higher content of Al_2_O_3_ has composed by amorphous Al_2_O_3_ and small size of crystalline NbB_2_ grains. The momentum transferred in electron collisions is less efficient to induce mass transport, as occurs the radiation enhanced diffusion by electrons is not enough to induce film densification, as can be verified by TEM results in Fig. 5, suggesting that this less dense structure permits the relaxation of the film with lower residual stress [24]. The change of structure in the lower content of Al_2_O_3_ makes the films’ residual stress increased because of the crystallization c-Al_2_O_3_ in the interface of large size crystalline NbB_2_ grains.

**Fig. 7:**
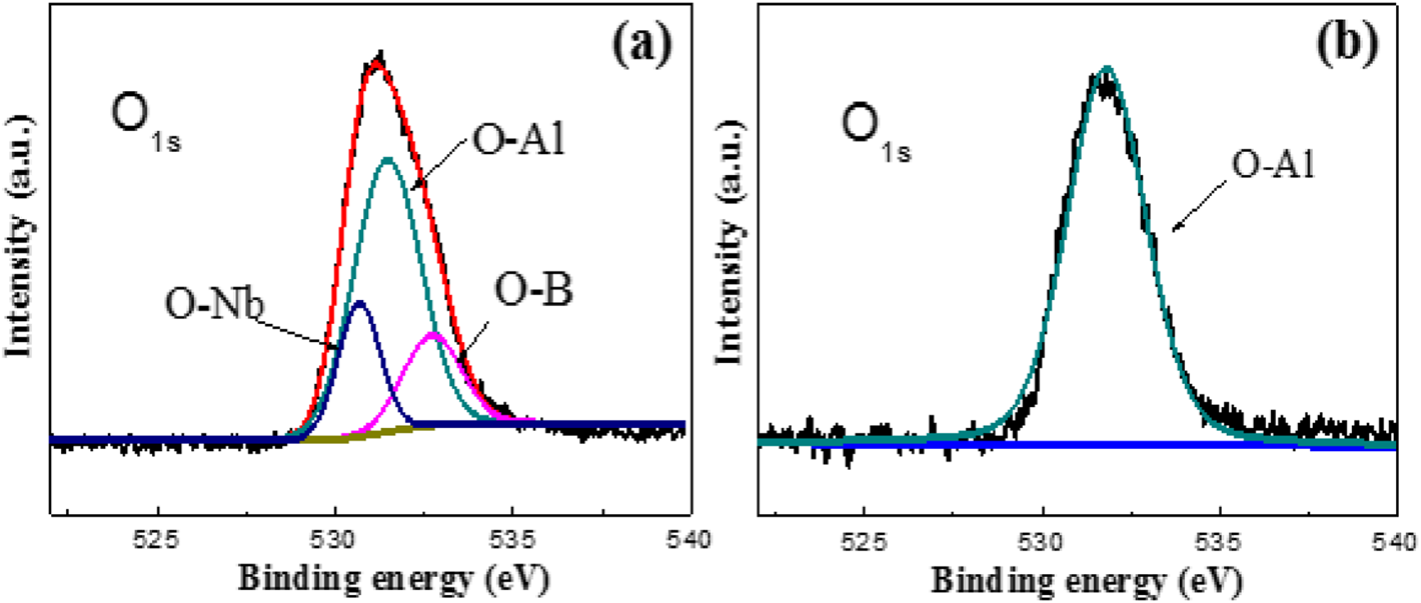
Nb-B-Al-O Nanocomposite films’ high-resolution XPS spectra of O_1s_ in 60 W power (**a**) and 120 W power (**b**) of Al_2_O_3_

From the analysis of different power of Al_2_O_3_’s structure, we choose two typical contents of Al_2_O_3_ (60 and 120 W) as example to explore its oxidation resistance properties. XPS O_1s_ spectra from different power of Al_2_O_3_ are presented in Fig. 7. The spectrum for low power of Al_2_O_3_ in Fig. 7a shows three features: the strongest peak at 531.7 eV assigned to O-Al bond and other peak at 530.7 and 533.0 eV assigned O-Nb and O-B chemistry bonds, respectively. The exits of O-Nb and O-B chemical bonds are due to the combination of NbB_2_ and Al_2_O_3_. Besides that, the film’s oxidation in air condition can also lead to this chemistry bonding. Since the content of Al_2_O_3_ is pretty small in the nanocomposite films (from Fig. 1), the chemistry bonding of O-Nb and O-B is also too little to find from XRD results. As the content of Al_2_O_3_ is decreased in the nanocomposite films, a slight shift is seen in the O_1s_ spectra, from about 531.2 eV for the low power of Al_2_O_3_ to about 531.7 eV for the high power of Al_2_O_3_(b). And, there only exits O-Al chemical bond, which means Al only exists in the form of Al_2_O_3_. This shift can be explained that by decreasing the content of Al_2_O_3_, it can change the oxidation resistance properties of nanocomposite film. When the content of Al_2_O_3_ is higher, the film shows a nanocomposite microstructure with amorphous Al_2_O_3_ embedded in weak NbB_2_ nanocrystalline phase. The weak crystallization of NbB_2_ means the B atoms and Nb atoms are easy to form chemistry bonds with O atoms. With the decreasing of Al_2_O_3_, the NbB_2_ shows good crystalline with large size and there appears to cubic-Al_2_O_3_ crystallization, so the O atoms are only formed by the Al-O bond. This is in agreement with the results from XRD and TEM.

**Fig. 8:**
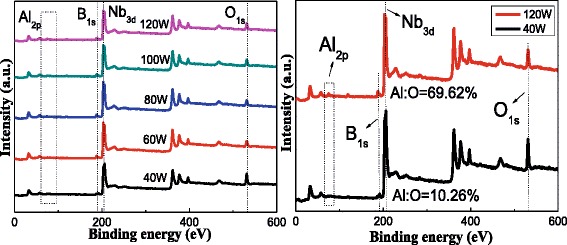
Nb-B-Al-O nanocomposite films’ XPS whole energy spectra of different power of Al_2_O_3_

Figure 8 shows the nanocomposite films’ XPS whole energy spectra of different power of Al_2_O_3_. The atomic concentration specific value of B and Nb is basically remaining stable. On the contrary, as the power of Al_2_O_3_ rises to 120 W, the atomic concentration specific value of Al and O is increased from 10.56 % in 40 W to 69.62 % in 120 W, and the main existing form in 120 W is Al-O bond. This can also be explained by the change of crystalline in NbB_2_ and Al_2_O_3_ phase and is confirmed with the results showed in XPS O_1s_ spectra. A general observation is that decreasing the content of Al_2_O_3_ can enhance the films’ oxidation resistance properties.

**Fig. 9:**
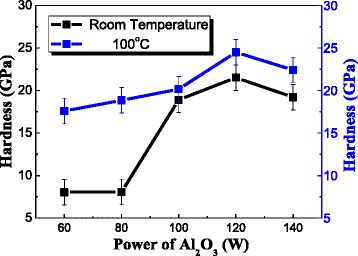
Hardness and elastic modulus with room temperature and 100 °C deposition temperature of Nb-B-Al-O nanocomposite films at different power of Al_2_O_3_

In order to explore the films’ thermostability properties, we test the hardness from room temperature to 300 °C with different power of Al_2_O_3_. With the increasing of temperature, the change trend of hardness is just the same. So we choose 100 °C to make a comparison. From Fig. 9, we can see that the influence of Al_2_O_3_’s content is pretty clear on the hardness of nanocomposite films at room temperature. But when the temperature is increased, the hardness change variation of different Al_2_O_3_ contents is reduced. The less content of Al_2_O_3_ can make this film remains the good mechanical properties and also a good thermostability.

## Conclusions

Nb-B-Al-O nanocomposite films were deposited on Si substrate by magnetron sputtering. The effect of Al_2_O_3_’s content on structure and properties were investigated. Decreasing the content of Al_2_O_3_ through increasing the power can appear cubic-crystallization of Al_2_O_3_ between large sizes of NbB_2_ grains. This structure change can enhance the mechanical properties and oxidation resistance properties of nanocomposite films and keep thermal stability. The maximum hardness and elastic modulus were up to 21.60 and 332.78 GPa at higher power of Al_2_O_3_. The change of interface structure between Al_2_O_3_ and NbB_2_ and theory of bombarding energy plays an important role in its properties. Because of crystallization of Al_2_O_3_’s less content, the mechanical properties can keep better oxidation resistance and stability at high temperature. Our results showed that the combined aluminum oxide and NbB_2_ can produce a positive effect on properties. Nb-B-Al-O films appear to be a promising nanocomposite system suitable for engineering applications.

## Abbreviation

Lc: load of critical fracture

## References

[1] Mayrhofer PH, Mitterer C, Wen JG, Greene JE, Petrov I (2005). Self-organized nanocolumnar structure in superhard TiB_2_ thin films. Appl Phys Lett.

[2] Cumberland RW, Weinberger MB, Gilman JJ, Clark SM, Tolbert SH, Kaner RB (2005). Osmium diboride, an ultra-incompressible, hard haterial. J Am Chem Soc.

[3] Latini A, Rau JV, Ferro D, Teghil R, Alberbini VR, Barinov SM (2008). Superhard rhenium diboride films: preparation and characterization. Chem Mater.

[4] Kalfagiannis N, Volonakis G, Tsetseris L, Logothetidis S (2011). Excess of boron in TiB_2_ superhard thin films: a combined experimental and ab initio study. J Phys D Appl Phys.

[5] Niu HY, Wang JQ, Chen XQ, Li DZ, Li YY, Lazar P (2012). Structure, bonding and possible superhardness of CrB_4_. Phys Rev B.

[6] Otani S, Korsukova MM, Mitsuhashi T (1998). Floating zone growth and high-temperature hardness of NbB_2_ and TaB_2_ single crystals. J Cryst Growth.

[7] Murakami T, Xu CN, Kitahara A, Kawahara M, Takahashi Y, Inui H, Yamaguchi M (1999). Microstructure, mechanical properties and oxidation behavior of powder compacts of the Nb-Si-B system prepared by spark plasma sintering. Intermetallics.

[8] Shein IR, Lvanovskii AL (2002). Band structure of ZrB_2_, VB_2_, NbB_2_, and TaB_2_ hexagonal diborides: comparison with superconducting MgB_2_. Phys Solid State.

[9] Kang YB, Li DJ, Wang HY, Yan JY, Zhang S, Gong J (2012). Growth, microstructure, and mechanical properties related to modulation period for ZrAlN/ZrB_2_ superlattice coatings. Appl Surf Sci.

[10] Sun YD, Li DJ, Gao CK, Wang N, Yan JY, Dong L, Cao M, Deng XY, Gu HQ, Wan RX (2013). The effect of annealing on hardness, residual stress, and fracture resistance determined by modulation ratios of TiB_2_/TiAlN multilayers. Surf Coat Technol.

[11] Wang S, Li Y, Zhang XH (2013). Influence of the microstructure evolution of ZrO_2_ fiber on the fracture toughness of ZrB_2_-SiC nanocomposite ceramics. Mater Des.

[12] Liu J, Ownby PD (1991). Enhanced mechanical properties of alumina by dispersed titanium diboride particulate inclusions. J Am Ceram Soc.

[13] Sundaram V, Logan KV, Speyer RF (1997). Aluminothermic reaction path in the synthesis of TiB_2_–Al_2_O_3_ composite. J Mater Res.

[14] Mishra SK, Bhople A, Paswan S (2014). Microstructure, hardness, toughness and oxidation resistance of Al_2_O_3_-ZrB_2_ composite with different Ti percentages prepared by in-situ SHS dynamic compaction. Int Journal of Refractory Metals and Hard Materials.

[15] Liu N, Dong L, Li XF, Li DJ, Wan RX, Gu HQ (2015). Controllable substrate bias voltages effectively tailoring nanocomposite Nb-B-Al-O film properties. J Alloys Compd.

[16] Janssen GCAM, Abdalla MM, Keulen FV, Pujada BR, Venrooy BV (2009). Celebrating the 100th anniversary of the Stoney equation for film stress: developments from polycrystalline steel strips to single crystal silicon wafers. Thin Solid Films.

[17] Oliver WC, Pharr GM (1992). An improved technique for determining hardness and elastic modulus using load and displacement sensing indentation experiments. J Mater Res.

[18] Li DJ, Tan M, Liu GQ, Liu MY, Deng XY, Liu H, Sun X (2010). The influence of N+ beam bombardment and deposition temperature on the growth of ZrB_2_/WNx nanomultilayers. Surf Coat Technol.

[19] Zhuang CQ, Schlemper C, Fuchs R, Zhang L, Huang N, Vogel M, Staedler T, Jiang X (2014). Mechanical behavior related to various bonding states in amorphous Si-C-N hard films. Surf Coat Technol.

[20] Nedfors N, Tengstrand O, Flink A, Eklund P, Hultman L, Jansson U (2013). Characterization of amorphous and nanocomposite Nb-Si-C thin films deposited by DC magnetron sputtering. Thin Solid Films.

[21] Mayrhofer PH, Sonnleitner D, Bartosik M, Holec D (2014). Structural and mechanical evolution of reactively and non-reactively sputtered Zr-Al-N thin films during annealing. Surf Coat Technol.

[22] Koehler JS (1994). Attempt to design a strong solid. Phys Rev B.

[23] Li DJ, Cao M, Deng XY (2007). Multilayered coatings with alternate ZrN and TiAlN superlattices. Appl Phys Lett.

[24] Sanchez CMT, Plata BR, Costa MEHM, Freire FLJ (2011). Titanium diboride thin films produced by dc-magnetron sputtering: structural and mechanical properties. Surf Coat Technol.

